# Culling Less Fit Neurons Protects against Amyloid-β-Induced Brain Damage and Cognitive and Motor Decline

**DOI:** 10.1016/j.celrep.2018.11.098

**Published:** 2018-12-26

**Authors:** Dina S. Coelho, Silvia Schwartz, Marisa M. Merino, Barbara Hauert, Barbara Topfel, Colin Tieche, Christa Rhiner, Eduardo Moreno

**Affiliations:** 1 Cell Fitness Lab, Champalimaud Centre for the Unknown, Av. Brasília, 1400-038 Lisbon, Portugal; 2 Institute for Cell Biology, University of Bern, Baltzerstrasse 4, 3012 Bern, Switzerland; 3 Stem Cells and Regeneration Lab, Champalimaud Centre for the Unknown, Av. Brasília, 1400-038 Lisbon, Portugal; 4 Department of Biochemistry, University of Geneva, Quai Ernest-Ansermet 30, 1211 Geneva 4, Switzerland

**Keywords:** cell fitness, neurodegeneration, apoptosis, β-amyloid, neuronal selection, Alzheimer’s, Drosophila, cell competition, azot

## Abstract

Alzheimer’s disease (AD) is the most common form of dementia, impairing cognitive and motor functions. One of the pathological hallmarks of AD is neuronal loss, which is not reflected in mouse models of AD. Therefore, the role of neuronal death is still uncertain. Here, we used a *Drosophila* AD model expressing a secreted form of human amyloid-β42 peptide and showed that it recapitulates key aspects of AD pathology, including neuronal death and impaired long-term memory. We found that neuronal apoptosis is mediated by cell fitness-driven neuronal culling, which selectively eliminates impaired neurons from brain circuits. We demonstrated that removal of less fit neurons delays β-amyloid-induced brain damage and protects against cognitive and motor decline, suggesting that contrary to common knowledge, neuronal death may have a beneficial effect in AD.

## Highlights

• Peptides linked to neurodegenerative diseases reduce neuronal fitness in *Drosophila*
• β-amyloid-induced neuronal death is mediated by fitness regulators *flower* and *azot*
• Suppression of fitness-based neuronal culling aggravates cognitive and motor decline
• Neuronal death related to fitness-based selection has a beneficial net effect

## Graphical Abstract

**Figure:**
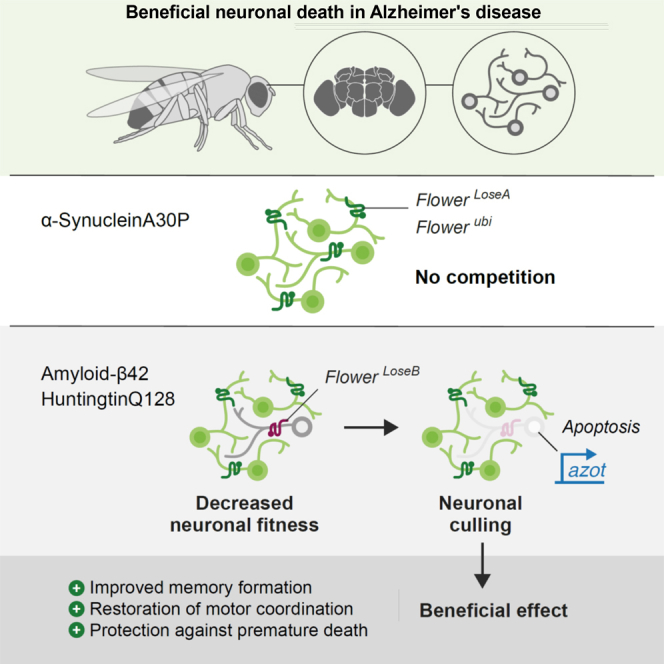

## Teaser

Multicellular organisms eliminate abnormal but viable cells based on their fitness status through cell competition to maintain tissue integrity. Here, Coelho et al. report that fitness-based neuronal selection occurs in the course of neurodegeneration. Death of unfit neurons is beneficial, protecting against disease progression by restoring motor and cognitive functions.

## Introduction

Multicellular organisms have evolved mechanisms to maintain tissue homeostasis and integrity throughout development and aging. Besides cell-intrinsic surveillance mechanisms, relative fitness levels within a cell population are constantly monitored, ensuring the removal of suboptimal but otherwise viable cells (Merino et al., 2016). The elimination of potentially dangerous or abnormal cells based on their fitness status is known as cell competition. Recent findings prove cell competition is a broad biological process proposed to constitute a quality control mechanism against developmental malformations (de la Cova et al., 2004, Gibson and Perrimon, 2005, Moreno et al., 2002), tumorigenesis (Alexander et al., 2004, Hogan et al., 2009, Kajita and Fujita, 2015, Martins et al., 2014, Menéndez et al., 2010), and aging (Merino et al., 2015). However, the cell competition machinery may be subverted by precancerous cells to acquire a super-fit status, enabling them to expand, kill, and invade surrounding wild-type tissue with a lower fitness status (Eichenlaub et al., 2016, Levayer et al., 2015, Moreno and Basler, 2004, Suijkerbuijk et al., 2016). Still, cell competition has not yet been investigated in the course of aging-associated disorders, particularly in neurodegenerative diseases.

In *Drosophila*, the fitness status is translated at the cellular level by fitness fingerprints, which are encoded by distinct isoforms of the Flower protein located at the extracellular membrane (Petrova et al., 2012, Rhiner et al., 2010, Yao et al., 2009). Flower is a conserved protein with three isoforms in *Drosophila* that differ solely at the extracellular C terminus: Flower^ubi^ is expressed ubiquitously, while Flower^LoseB^ and Flower^LoseA^ are upregulated in suboptimal cells. The display of loser isoforms in a subset of cells is sufficient to target them for elimination by apoptosis, which depends on the transcription of the fitness checkpoint gene *azot* (Merino et al., 2015). Azot is an EF-hand calcium binding protein dedicated exclusively to cell competition-related apoptosis that integrates upstream relative fitness levels and targets suboptimal cells for death and subsequent engulfment by hemocytes (Portela et al., 2010, Casas-Tintó et al., 2015, Lolo et al., 2012). Mounting evidence demonstrates cell competition is a conserved process ranging from *Drosophila* to mammals that can also occur in post-mitotic cells and differentiated adult tissue such as follicular epithelia or the neural system (Kolahgar et al., 2015, Tamori and Deng, 2013). The cell competition mediators *flower* and *azot*, for example, have been found to mediate elimination of injured or misconnected neurons (Merino et al., 2013, Moreno et al., 2015). The flower code is cell type specific; in the nervous system, only Flower^LoseB^, not Flower^LoseA^, is expressed in suboptimal neurons (Merino et al., 2013).

Neuronal loss is a key symptom of Alzheimer’s disease (AD), the most prevalent neurodegenerative disorder. AD is a slow, progressive disease characterized by initial subtle memory problems that deteriorate to severe cognitive impairment, behavioral changes, and difficulty to walk. The main pathological hallmarks of AD are brain deposition of extracellular amyloid plaques and intracellular fibrils of hyperphosphorylated tau, exacerbated inflammation, and finally neuronal damage and death (Braak and Braak, 1991). According to the amyloid cascade hypothesis, β-amyloid-related toxicity is considered the primary cause of the disease, but the mechanisms mediating amyloid-induced neurodegeneration and cognitive decline have not been fully elucidated (Ashe and Zahs, 2010, Huang and Mucke, 2012, Karran and De Strooper, 2016, Soldano and Hassan, 2014).

Post-mortem brain sections and structural MRI in AD patients show cerebral atrophy in regions involved in memory processing, such as the cortex and the hippocampus (Ossenkoppele et al., 2015, Seab et al., 1988). These findings suggest that the subpopulations of neurons primarily affected by AD, including the entorhinal cortex and the hippocampal CA1 projection neurons, may be more vulnerable to cellular stress responses elicited by misfolded amyloid (Gómez-Isla et al., 1996, Saxena and Caroni, 2011, Wakabayashi et al., 1994). Although central to human pathology, mechanisms of neuronal loss have been understudied *in vivo*, because AD mouse models do not recapitulate this aspect, showing little neuronal death (Ashe and Zahs, 2010, Karran et al., 2011).

Here we sought to analyze a potential role of fitness-based neuronal elimination in the context of AD onset and progression in a *Drosophila* model in which human β-amyloid expression is induced in the adult fly brain. We found a physiological mechanism that identifies and purges less fit neurons, delaying cognitive decline and motor disability.

## Results

### Expression of Amyloid-β42 in the *Drosophila* Nervous System Affects Neuronal Fitness

**Figure 1:**
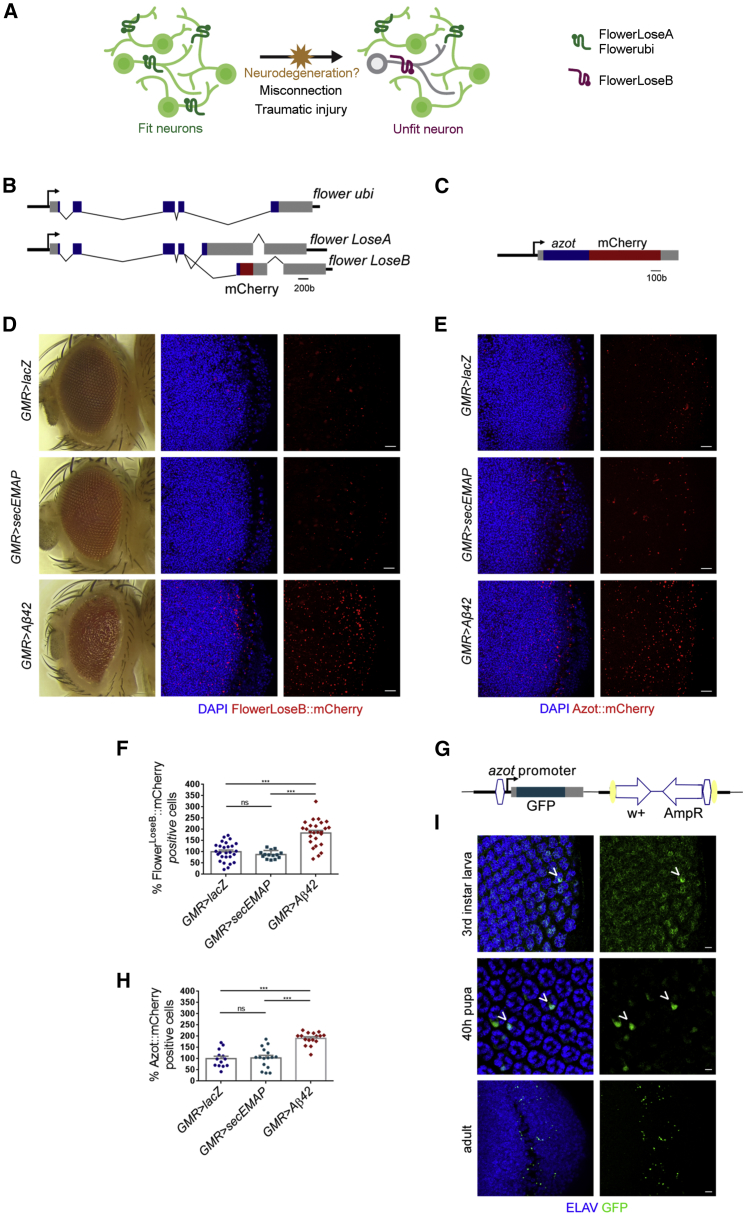
Expression of Amyloid-β42 in the *Drosophila* Nervous System Generates Suboptimal Neurons that Upregulate *flower*^*LoseB*^ and *azot* (A) Schematic illustrating neuronal fitness comparison. Neurons express ubiquitously Flower^ubi^ or Flower^LoseA^ at their cellular membrane, but external insults such as a traumatic injury or failing to establish synaptic connections during development can decrease the fitness status of one neuron (or one subpopulation of neurons) that upregulate Flower^LoseB^. We hypothesize that in the course of neurodegeneration induced by β-amyloid, neurons enter a period of suboptimality, characterized by a low fitness status and upregulation of the Flower^LoseB^ isoform. (B) Representation of the *flower*^*LoseB*^*::mCherry* reporter. Each *flower* isoform has a different last exon. Based on this particularity, we generated a reporter specific for *flower*^*LoseB*^ by introducing the mCherry sequence at the end of the exon specific for this isoform (exon 6). Blue rectangles are exons, the 5′ and 3′ UTRs are shown in gray, and the red box shows the localization of the mCherry tag (not to scale). (C) Schematic of the *azot::mCherry* reporter that was obtained by fusion PCR. This construct includes 2,430 bp of the *azot* promoter region, the *azot* exon plus 175 bp of the 3′ end fused to mCherry (in red). The azot coding region is in blue, and UTRs are represented in gray. (D) *flower*^*LoseB*^*::mCherry* reporter (red) is strongly upregulated in the optic lobe of *GMR >* Aβ42 (amyloid-β42) adults, but not in the optic lobe of *GMR>lacZ* or *GMR > secEMAP* controls of the same age; the nuclear marker DAPI is shown in blue. Scale bar: 10 μm. The eye of *GMR >* Aβ42 flies shows a strong degenerative phenotype. (E) *azot::mCherry* reporter (red) expressed in the optic lobe of adult flies in the presence of *GMR*-driven *lacZ*, *secEMAP*, or *Aβ42*; DAPI is shown in blue. Scale bar: 10 μm. (F) Quantification of the percentage of Flower^LoseB^::mCherry-positive cells in the optic lobes of the indicated genotypes. The number of Flower^LoseB^::mCherry-positive cells detected for the *GMR>lacZ* control group was assumed to be 100%. (G) Schematic of the modified *azot{KO;GFP}* locus. This transgenic line was generated by integration of a knockin construct containing the GFP sequence, under the control of the *azot* endogenous promoter, into the *azot* knockout locus. The 5′ and 3′ UTRs of the *azot* gene are shown in gray. The vector backbone was conserved in the knockin line (*w*+, *AmpR*). The yellow ellipses are *loxP* sites, and the white hexagons are *attL* regions. (H) Quantification of the percentage of Azot::mCherry-positive cells in the optic lobe of the indicated genotypes. The number of Azot::mCherry-positive cells for the *GMR>lacZ* control group was assumed to be 100%. (I) Eye imaginal discs of third-instar larva, retina of 40-hr pupa, and adult optic lobes of *GMR > Aβ42* adults showing immunolabeling for the nuclear marker ELAV (blue) and endogenous GFP signal produced from *azot{KO;GFP}* (green). Arrowheads point to co-localization between ELAV and GFP. Scale bar: 5 μm. Error bars show SEM. Ns, no significant. ^∗∗∗^p < 0.001. All genotypes are heterozygous. See also Figures S1 and S2.

First, we tested whether neurons transit through a stage of reduced fitness when overexpressing Aβ42 (Figure 1A). We expressed a cassette containing two copies of the human amyloid-β42 (Aβ42) peptide fused to a signal peptide for secretion, under the control the *GMR-Gal4* driver, known to produce a strong degenerative phenotype in the *Drosophila* eye (Figure 1D) (Casas-Tinto et al., 2011), henceforth abbreviated as *GMR > Aβ42*. To monitor cell fitness markers in the optic lobe, where *GMR-Gal4* is expressed, we devised a sensitive reporter to detect Flower^LoseB^ by knocking in a *flower*^*LoseB*^*::mCherry*-tagged construct in the endogenous *flower* locus (Figure 1B). Flower^LoseB^*::mCherry* (indicator of low fitness) was strongly upregulated in the adult optic lobe of *GMR > Aβ42* flies, but not in the *GMR>lacZ* control (Figures 1D and 1F).

To control whether the secretion of a peptide is sufficient to downregulate fitness levels and induce *flower*^*LoseB*^, we expressed the secreted form of a small peptide, endothelial monocyte-activating polypeptide (EMAP) (17 kDa), under the control of *GMR-Gal4*. Secreted EMAP is a chemotactic clue that attracts hemocytes to sites of cell competition (Casas-Tintó et al., 2015). We confirmed that secretion of EMAP alone does not upregulate Flower^LoseB^*::mCherry* in the optic lobe, indicating that secretion of an innocuous peptide is not sufficient to decrease the fitness levels of neurons (Figures 1D and 1F).

The Flower^LoseB^ isoform was particularly upregulated in neurons of the optic lobes, as detected with the neuronal marker Elav (Figure S1A). Accordingly, Flower^LoseB^ expression did not co-localize with cells expressing the glial marker Repo (Figure S1B).

**Figure 2:**
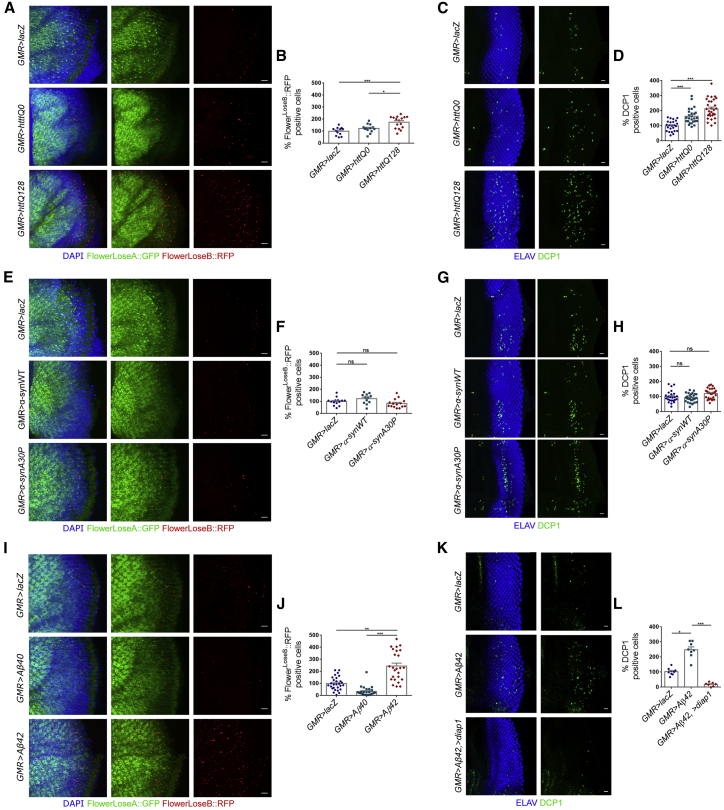
Ectopic Expression of HuntingtinQ128, but Not α-SynucleinA30P, Induces an Upregulation of the Flower^LoseB^ Isoform (A and B) Expression of the *flower*^*ubi*^*::YFP, flower*^*LoseA*^*::GFP, flower*^*LoseB*^*::RFP* reporter in the optic lobe of adult flies (A), and quantification (B) of the RFP-positive cells (red) for the following genotypes: *GMR>lacZ*, *GMR>httQ0*, and *GMR>httQ128*. DAPI is shown in blue, and GFP is shown in green. Scale bar: 10 μm. (C and D) Quantification (D) of the number of apoptotic cells in the eye discs of third-instar larva expressing *UAS-lacZ*, *UAS-httQ0*, or *UAS-httQ128* under the control of *GMR-Gal4* driver, and representative figures for each genotype (C). Apoptotic cells are marked by DCP1 in green, and nuclei are shown in blue. Scale bar: 10 μm. (E and F) Expression of the *flower*^*ubi*^*::YFP, flower*^*LoseA*^*::GFP, flower*^*LoseB*^*::RFP* reporter in the optic lobe of adult flies (E), and quantification (F) of RFP-positive cells (red) for the following genotypes: *GMR>lacZ*, *GMR > α-synWT*, and *GMR > α-synA30P*. DAPI is shown in blue, and GFP is shown in green. Scale bar: 10 μm. (G and H) Quantification (H) of the number of apoptotic cells in the eye discs of third-instar larva expressing *UAS-*l*acZ*, *UAS-*α-*synWT*, or *UAS-*α-*synA30P* under the control of *GMR-Gal4*, and representative figures (G) for each genotype. Apoptotic cells are marked by DCP1 in green, and nuclei are represented in blue. Scale bar: 10 μm. (I and J) Expression of the *flower*^*ubi*^*::YFP, flower*^*LoseA*^*::GFP, flower*^*LoseB*^*::RFP* reporter in the optic lobe of adult flies (I), and quantification (J) of RFP-positive cells (red) for the following genotypes: *GMR>lacZ*, *GMR > Aβ40*, and *GMR > Aβ42*. DAPI is shown in blue, and GFP is shown in green. Scale bar: 10 μm. (K and L) Quantification (L) of the number of apoptotic cells in the eye discs of third-instar larva expressing *UAS-lacZ*, *UAS-Aβ42*, or *UAS-Aβ42/UAS-diap1* under the control of *GMR-Gal4*, and representative figures for each genotype (K). Apoptotic cells are marked by DCP1 in green, and nuclei are represented in blue. Scale bar: 10 μm. Error bars indicate SEM. ^∗^p < 0.05, ^∗∗^p < 0.01, ^∗∗∗^p < 0.001. The number of positive cells for the genotype *GMR>lacZ* was assumed to be 100% for normalization. See also Figure S2.

We then tested activation of another marker of low fitness, *azot*, which is transcribed in cells destined to die based on previous fitness comparison (Merino et al., 2015). To visualize *azot* expression, we generated (1) *azot::mCherry* transgenic flies, which carry an extra copy of *azot* fused to *mCherry*, inserted in another chromosome (Figure 1C), and (2) *azot{KO;GFP}* flies, wherein *GFP* was placed in the endogenous locus of the previously knocked out *azot* gene (Figure 1G). With both lines, we found that *azot* not only was upregulated in the optic lobes of *GMR > Aβ42* adult flies but was already activated in neurons at previous developmental stages, including the eye discs of the larva and retinas of mid-pupa (Figures 1E, 1H, and 1I).

### Expression of Misfolding-Prone Toxic Peptides Linked to Huntington’s Disease, but Not to Parkinson Disease, Triggers Neuronal Competition

To investigate neuronal fitness comparison in other types of neurodegenerative diseases, we turned to published human transgenes reported to induce degenerative phenotypes in the fly: HuntingtinQ128 (HttQ128) and α-SynucleinA30P (α-SynA30P). HttQ128 is a pathogenic form of the human *huntingtin* gene that encodes an expanded repeat of 128 poly-glutamines, causing reduction of viability, retinal death, and abnormal motor behavior in *Drosophila* (Lee et al., 2004). α-SynA30P is a mutant allele linked to familial Parkinson disease that originates premature loss of dopaminergic neurons, formation of brain inclusions similar to Lewis bodies, and decrease of climbing ability when expressed in flies (Feany and Bender, 2000, Song et al., 2017). HttQ0 and α-SynWT, which carry a non-pathogenic form of *huntingtin* and the wild-type allele of *α-synuclein*, respectively, served as controls.

For this experiment, we employed a previously published translational reporter, in which Flower^UBI^, Flower^LoseA^, and Flower^LoseB^ are tagged with a specific fluorescent protein: yellow fluorescent protein (YFP), GFP, and red fluorescent protein (RFP), respectively (Yao et al., 2009). We discovered that expression of HttQ128 from the *GMR* driver induces augmented levels of Flower^LoseB^ in the adult brain, contrary to the non-pathogenic form, HttQ0 (Figures 2A and 2B). Surprisingly, levels of Flower^LoseB^ did not change with ectopic expression of the Parkinson-related peptides α-SynA30P and α-SynWT (Figures 2E and 2F). The same results were obtained using the Flower^LoseB^::mCherry reporter to detect changes in cell fitness levels upon expression of these toxic peptides in the eye imaginal disc of the larva (Figures S2A–S2C).

Although both HttQ128 and α-SynA30P induce neurodegeneration by accumulation of protein aggregates in *Drosophila* models, our results indicate that only HttQ128 triggers neuronal competition. This result may be explained by the difference in toxicity levels imposed on the tissue by each of these transgenes. We observed that HttQ128 expression leads to increased cell death in a larval epithelium (eye disc), in opposition to the α-SynA30P transgene, which did not lead to significantly increased apoptosis under the same conditions (Figures 2C, 2D, 2G, and 2H).

Accumulation of amyloid peptides in the brain is thought to be the first step in Alzheimer’s pathogenesis. Whereas Aβ42 is the main component of amyloid plaques found in human patients, Aβ40 is a shorter isoform of the human amyloid peptide that is less amyloidogenic. Aβ40 does not deposit as soluble oligomeric aggregates in *Drosophila* and does not cause neurodegeneration *in vivo* (Iijima et al., 2004, Speretta et al., 2012). When overexpressed in the *Drosophila* brain using *GMR-Gal4*, Aβ40 did not alter the levels of the Flower^LoseB^ reporter (Figures 2I–2L). We decided to focus on Aβ42-associated toxicity from now on because the levels of *azot* and *flower* were most affected by this peptide.

### Aβ42-Producing Clones Are Eliminated over Time from a Neuronal Epithelium

**Figure 3:**
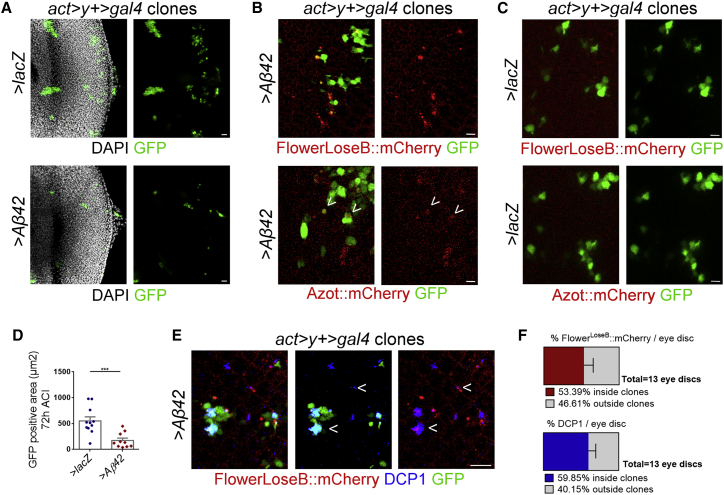
Amyloid β42-Producing Clones Are Eliminated over Time from a Neuronal Epithelium (A) Clones induced by heat shock of the flip-out cassette *act > y+ > gal4,UAS-GFP* in eye imaginal discs of third-instar larva. Clones are marked by GFP (green) and express *UAS-lacZ* or *UAS-Aβ42* 72 hr after clone induction (ACI). DAPI is in white. Scale bar: 20 μm. (B) Expression of the Flower^loseB^::mCherry reporter (red) 48 hr ACI or of the Azot::mCherry reporter (red) 72 hr ACI in *Aβ42*-overexpressing clones (green). Arrowheads indicate co-localization. Scale bar: 5 μm. (C) Clones (green) induced by heat shock of the flip-out cassette *act > y+ > gal4* driving *UAS-lacZ* in the eye imaginal disc of third-instar larva. No expression of the Flower^loseB^::mCherry reporter (red) or the Azot-mCherry reporter (red) is detected 48 or 72 hr ACI, respectively. Scale bar: 5 μm. (D) Quantification of GFP-positive area in *flip-out* clones overexpressing *lacZ* or *Aβ42* 72 hr ACI. (E) Detection of cleaved DCP1 (blue) and Flower^LoseB^::mCherry (red) expression in *act > y+ > gal4,UAS-GFP* clones (green) driving Aβ42 secretion 48 hr ACI. Arrows indicate co-localization. Scale bar: 10 μm. (F) Quantification of the percentage of Flower^LoseB^::mCherry signal or DCP1-positive signal localizing inside or outside *act > y+ > gal4* clones expressing Aβ42 48 hr ACI. See also Figure S2.

To determine whether Aβ42 induces cell elimination when expressed in clones, we induced its expression by heat shock in clones marked by GFP in the neuroepithelium of the eye disc. We registered that Aβ42-producing clones are progressively excluded from the tissue and detected a higher proportion of dying cells marked by *Drosophila* caspase-protein 1 (DCP1) inside these clones (Figures 3A and 3D–3F). Flower^LoseB^::mCherry and Azot::mCherry were mainly detected inside Aβ42-producing clones, but some signal was present outside clone borders (Figures 3B, 3C, 3E, and 3F). We found that Aβ42 largely diffused out of clone borders and accumulated at the basal side of the eye discs, explaining non-autonomous induction of *flower* and *azot* (Figure S2D). Flower^LoseB^::mCherry and Azot::mCherry were not detected in control clones expressing an innocuous transgene (Figure 3C). As expected, the cleaved form of DCP1 co-localized with Flower^LoseB^::mCherry, showing that unfit cells affected by Aβ42 were undergoing apoptosis (Figure 3E; Figure S2E).

### *flower* and *azot* Are Necessary for Aβ42-Induced Neuronal Death

**Figure 4:**
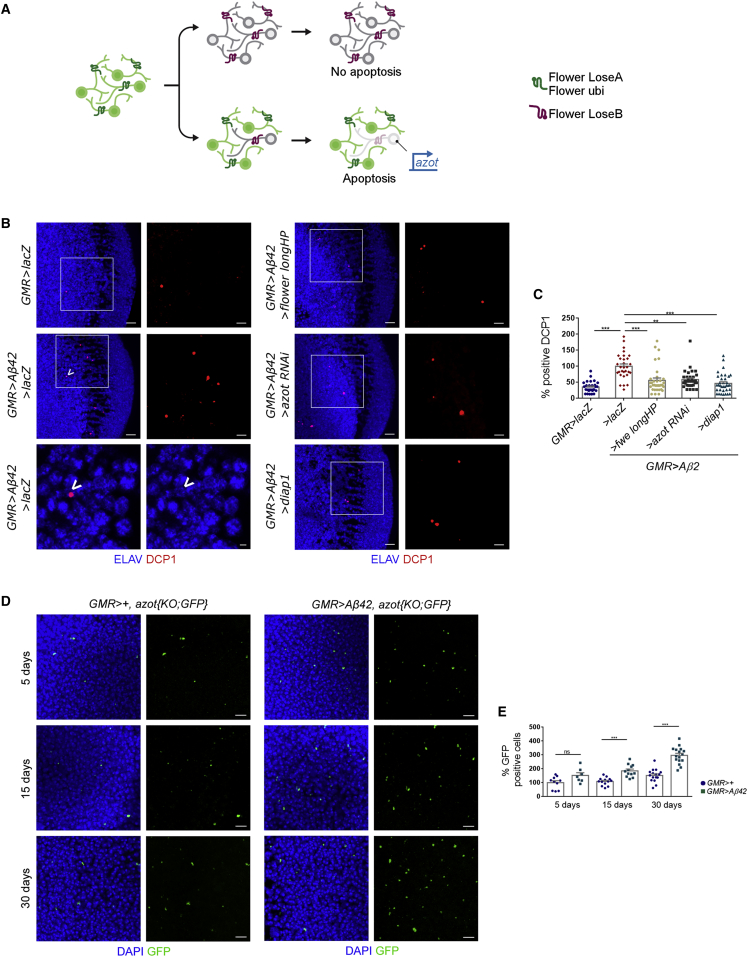
*flower* and *azot* Are Necessary for Amyloid-β42-Induced Neuronal Death (A) Neurons compute relative levels of Flower^LoseB^ to determine its fate. Neurons expressing LoseB and surrounded by neurons with equal levels of LoseB do not activate *azot* transcription. If the neuron cannot cope with an insult and exhibits persistently higher levels of LoseB compared to neighboring neurons, it will fail to pass a fitness checkpoint mediated by the *azot* gene and will be purged from the tissue. Increased copies of *azot* enhance fitness-based cell elimination. (B) Optic lobes of 2-week-old adults showing apoptotic neurons labeled by DCP1 (*Drosophila* caspase-protein 1) in red and ELAV in blue. Scale bar: 10 or 5 μm in the insets. At the bottom on the left panel is a dying neuron (arrow) from a single plane of the confocal projection displayed earlier, representative of the *GMR > Aβ42 / > lacZ* genotype. Scale bar: 2 μm. (C) Quantification of positive DCP1, assuming the levels of apoptosis in *GMR > Aβ42 / > lacZ* are 100%. (D) Tracing of suboptimal cells (green) through aging using the *azot{KO;GFP}* reporter in *GMR > + flies* or *GMR > Aβ42 flies* at 5, 15, or 30 days of life. DAPI marks cell nuclei (blue). Scale bar: 5 μm. (E) Quantification of GFP-positive cells per optic lobe in *GMR > +* flies or *GMR > Aβ42* flies at 5, 15, or 30 days of life. Error bars show SEM. Ns: no significant. ^∗∗^p < 0.01, ^∗∗∗^p < 0.001. All genotypes are heterozygous. See also Figure S3.

Next, we analyzed Aβ42-associated toxicity and neuronal loss in the adult brain. *GMR*-driven Aβ42 induces cell death in the optic lobe over time, eliciting a 2.8-fold increase in the number of positive cells for activated DCP1, which co-localized with the neuronal marker ELAV, compared to control flies 2 weeks after eclosion (Figures 4B and 4C).

The presence of Flower^LoseB^ isoforms at the cell membrane of a particular neuron does not imply that the cell will die (Merino et al., 2013, Moreno et al., 2015, Rhiner et al., 2010) (Figure 4A). Cell death is initiated only if relative fitness differences with neighboring neurons persist (Levayer et al., 2015, Merino et al., 2013, Rhiner et al., 2010), which requires downstream transcriptional activation of *azot* (Merino et al., 2015) (Figure 4A).

To check whether neuronal fitness comparisons mediate Aβ42-induced death, we modulated *flower* and *azot* genetic dosages. We found that suppressing relative differences of Flower^LoseB^ levels among cells by silencing *LoseA/B* isoforms via a long hairpin (Merino et al., 2013) is sufficient to induce a strong decrease in total apoptosis detected upon Aβ42 expression in the adult brain, bringing it back to almost wild-type levels (Figures 4B and 4C). Silencing *azot* with RNAi also reduced the cell death observed in the presence of Aβ42 alone, bringing it back to almost wild-type levels (Figures 4B and 4C).

As a positive control for inhibition of apoptosis, we used *UAS-dIAP1* (*>dIAP1*), an antagonist of the apoptotic pathway in *Drosophila* (Hay et al., 1995). Bulk cell death in *GMR > Aβ42* adult optic lobes was suppressed by overexpression of dIAP1 (*Drosophila* inhibitor of apoptosis 1) (Figures 4B and 4C). We confirmed that part of apoptotic cells marked by DCP1 co-localize with Azot::mCherry in a *GMR > Aβ42* background (Figure S1C). These results were supported by an equivalent experiment conducted in the eye imaginal disc of the larva (Figures S3A and S3B), showing that Aβ42-associated cell death is mediated by *flower* and *azot*. Moreover, we found that the fitness-based neuronal elimination induced by neurodegeneration is not specific to *Aβ42* and occurs in HttQ128-associated degeneration (Figures S3C and S3D).

To study how neuronal fitness is affected over time, we monitored cumulative *azot* expression during aging of *GMR > Aβ42* brains with the reporter line *azot{KO;GFP}*. This reporter allows visualization of impaired cells (GFP+) that activate the *azot* promoter (Merino et al., 2015). Using this tool, we observed that GFP signal is only detected in Flower^LoseB^-positive cells (Figure S1D). Optic lobes overexpressing Aβ42 accumulate GFP-positive cells at an increased rate compared to control brains of identical ages lacking Aβ42 (53%, 70%, and 96% increase over the wild-type of the same age at 5, 15, and 30 days, respectively) (Figures 4D and 4E). Altogether, this shows that Aβ42 expression leads to a progressive generation of neurons that will be targeted to death via *azot* (Figure 4A).

### Suppression of Fitness-Based Removal of Aβ42-Damaged Neurons Aggravates Accumulation of Degenerative Vacuoles and Decreases Lifespan

**Figure 5:**
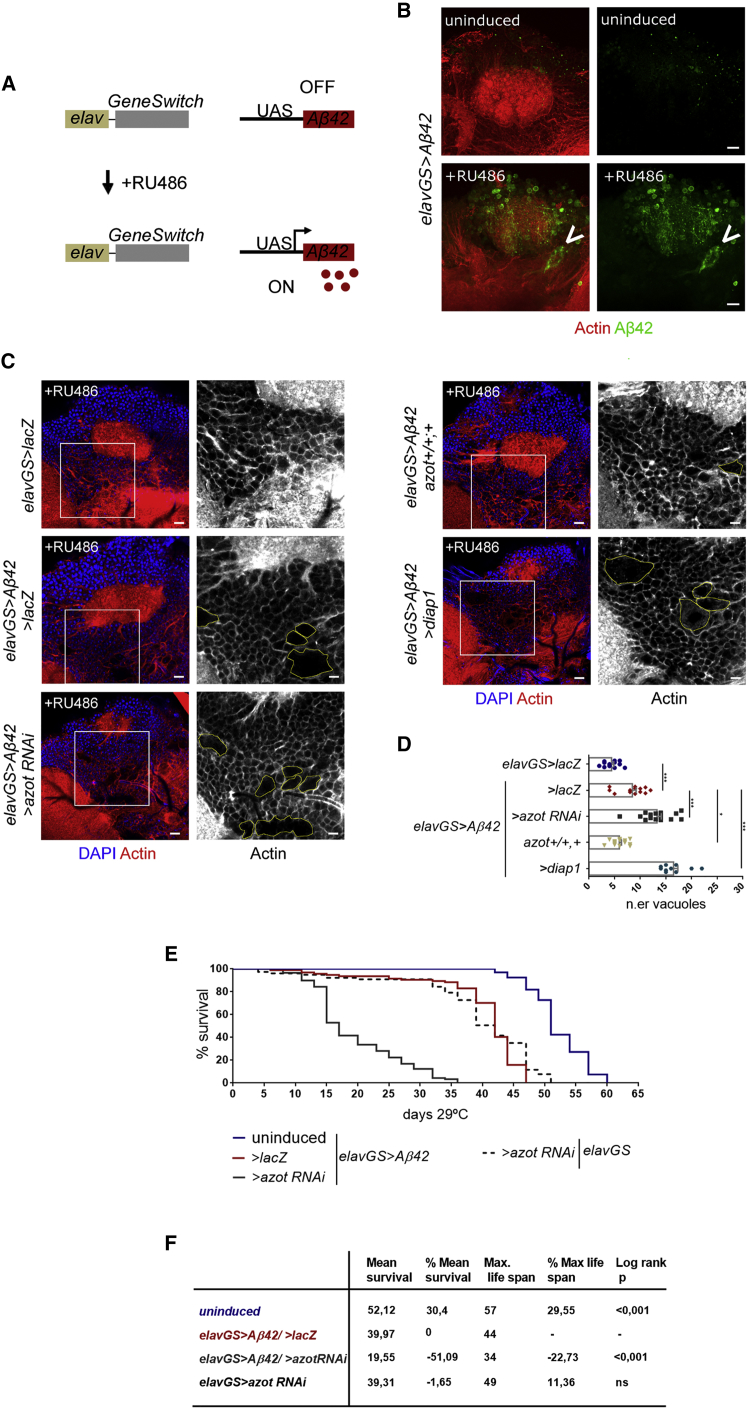
Suppression of Fitness-Based Neuronal Selection Decreases Lifespan in a *Drosophila* Model of AD (A) *GeneSwitch* system relies on a chimerical Gal4 containing a steroid receptor domain that only becomes active upon binding the synthetic progesterone analog RU486. This system allows us to induce the conditional expression of the human Aβ42 peptide in the adult brain of *elav-GeneSwitch* (*elavGS*) transgenic flies upon addition of RU486 to the food. (B) Aβ42 (green) expression is detected in the brain of RU486-induced *elavGS > Aβ42* flies, but not in the brain of uninduced flies. Aβ42 can form large insoluble aggregates (arrowhead). A posterior view of the brain next to the mushroom body calyx is shown. Actin is in red. Scale bar: 5 μm. (C) Posterior view of the brain, showing the surrounding region of the mushroom body calyx. Nuclei are marked by DAPI (blue), and actin cytoskeleton is highlighted by phalloidin (red). Degenerative vacuoles are surrounded by a yellow line in grayscale insets. All genotypes were treated with RU486. Scale bar: 10 μm in color pictures or 5 μm in grayscale insets. (D) Mean number of vacuoles located at a 10-μm-deep plan in 2-week-old brains of the indicated genotypes. Data are represented as mean ± SEM. ^∗∗∗^p < 0.001, ^∗∗^p < 0.01, ^∗^p < 0.05. (E and F) Lifespan curve (E) and table (F) depicting survival analysis for heterozygous females of the following genotypes: uninduced *elavGS >* Aβ42 / > *lacZ*, induced elavGS > *Aβ42 / > lacZ*, induced *elavGS > Aβ42 / > azot* RNAi, and induced *elavGS > azot* RNAi. See also Figures S4 and S5.

Next, we asked about the consequences of blocking fitness-based elimination of Aβ42-damaged cells for aging, locomotion, and cognition. First, we established a model in which expression of *Aβ42* is restricted to adult neurons; to this end, we generated flies containing the *Aβ42* cassette under the control of the inducible promoter *elav-GeneSwitch* (*elavGS*) (Figure 5A) (Poirier et al., 2008, Roman et al., 2001). We detected Aβ42 accumulation in the optic lobe and mushroom body calyx of *elavGS > Aβ42* adult flies fed for 5 days on the *GeneSwitch*-activator RU486, but not in the brain of uninduced flies (Figure 5B; Figure S4A). Aβ42 aggregates stained positive for aggresome markers (Figure S4B), confirming the amyloidogenic nature of human Aβ42 when secreted by *Drosophila* neurons.

Apoptosis was increased in the optic lobes of 10-day-old adults raised on RU486 (Figure S4G) compared to uninduced flies. TUNEL-positive cells co-localized with ELAV, indicating that Aβ42 caused neuronal death (Figure S4G). RU486 did not cause apoptosis on its own (Figures S4C and S4D). Increased cell death was not accompanied by elevated proliferation, consistent with little regeneration occurring in uninjured adult brains (Fernández-Hernández et al., 2013) (Figures S4E and S4F).

Brains of induced *elavGS > Aβ42* flies showed hallmarks of neurodegeneration, such as increased number of degenerative vacuoles (Figures 5C and 5D). In induced *elavGS > Aβ42* flies, the total number of vacuoles was the double that of *elavGS>lacZ* control flies of the same age (Figures 5C and 5D). *azot* knockdown in induced *elavGS > Aβ42* aggravated brain degeneration and caused a 57% increase in the total number of neurodegenerative vacuoles (Figures 5C and 5D) Conversely, when induced *elavGS > Aβ42* flies were provided with a third functional copy of *azot*, which is known to accelerate the elimination of unfit cells (Merino et al., 2015), brain architecture was restored and the number of vacuoles dropped 30% (Figures 5C and 5D). Finally, we suppressed apoptosis by overexpressing dIAP1, together with Aβ42, in adult neurons and observed that brains deteriorated faster than in induced *elavGS > Aβ42* flies alone (Figures 5C and 5D).

To rescue brain morphology, we used the *azot{KO;hid}* transgenic line, which contains the coding sequence of the pro-apoptotic gene *hid* inserted into the *azot KO* locus, leading to *hid* transcription under the control of *azot* endogenous enhancer sequences (Merino et al., 2015). The total size of vacuoles in the brain of induced *elavGS > Aβ42/ azot{KO;hid}* flies, which lack Azot protein but still eliminate unfit cells via transcription of *hid*, was significantly decreased at 2 weeks, proving *azot* has a role mainly dedicated to apoptosis regulation in the course of neurodegeneration (Figures S5A and S5B).

The observation that suppression of apoptosis led to accelerated vacuole formation in the brain made us speculate that cells may be undergoing alternative forms of cell death, such as necrosis. To test this hypothesis, we followed a protocol using propidium iodide (PI) that can penetrate compromised membranes of necrotic cells (Liu et al., 2014, Yang et al., 2013). We detected an increased number of cells permeable to PI in the brains of *elavGS > Aβ42* flies two weeks after induction, comparing to non-induced flies, indicating that Aβ42 can trigger necrosis in the brain (Figure S5C). However, blocking apoptosis by either overexpression of dIAP1 or knockout of *azot* did not lead to a further increase in the levels of necrosis, making necrosis an unlikely cause of accelerated vacuole formation in these genotypes (Figure S5C).

When analyzing life expectancy of *elavGS > Aβ42* flies, we confirmed that secreted Aβ42 is detrimental for longevity (Figures 5E and 5F): induced flies lived on average 40 days versus 52 days for uninduced flies. Life expectancy of induced *elavGS > Aβ42* flies dropped to 20 days when *azot* expression was silenced by RNAi (representing a 51% decrease in mean survival compared with *elavGS > Aβ42* flies carrying a wild-type dose of *azot*) (Figures 5E and 5F). It was not possible to determine a clear effect of *diap1* overexpression on longevity or in combination with Aβ42 (data not shown). Apoptosis is involved in many biological processes with potentially opposing consequences for lifespan.

### An Extra Copy of *azot* Is Sufficient to Restore Motor Coordination and Improve Long-Term Memory Formation

**Figure 6:**
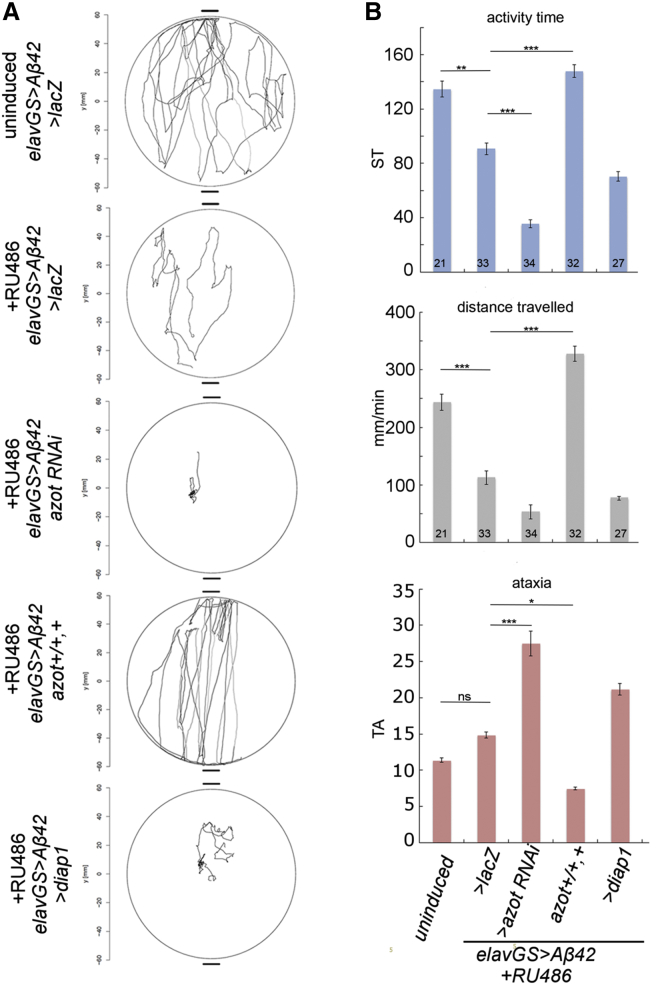
Extra Copy of *azot* Is Sufficient to Restore Motor Coordination in *AD* Flies (A) Individual walking trajectories (5 min tracking) representative of each indicated genotype. (B) Graphs depict activity time in seconds (using a speed threshold [ST]) (Colomb et al., 2012), distance walked in millimeters per minute, and median turning angle (TA) in degrees. Parameters were calculated from individual walks of 2-week-old heterozygous flies raised on RU486 for each genotype. Statistical significance was based on ANOVA. All genotypes were compared to one another. See also Figure S6 and Videos S1, S2, and S3.

We studied the consequences of fitness-based neuronal culling on walking behavior. Using tracking software (Colomb et al., 2012), we extracted several behavioral parameters from 5 min walking sessions of individual flies (Figure 6A). *elavGS > Aβ42* flies induced for two weeks on RU486 showed decreased activity time, shorter walks, and ataxia (Figure 6B) compared to uninduced *elavGS > Aβ42* flies (Videos S1 and S2). Expression of *azot* RNAi significantly exacerbated behavioral and locomotor dysfunctions caused by Aβ42 alone (Figures 6A and 6B). On the contrary, an extra copy of *azot* was sufficient to restore the behavioral defects observed in *elavGS > Aβ42* flies, including lengths of walks, activity time, and ataxia (Figures 6A and 6B; Video S3). Finally, blocking apoptosis with *UAS-diap1* in *elavGS > Aβ42* individuals compromised walking performance (Figures 6A and 6B), whereas dIAP1 overexpression alone did not result in impaired locomotion (Figure S6A).

Video S1. Locomotion of an Uninduced *elavGS > Aβ42 / > lacZ* Individual, Related to Figure 6 Walking trajectories of heterozygous e*lavGS > Aβ42/+* females two weeks after eclosion on normal food.

Video S2. Locomotion of an Induced *elavGS > Aβ42 / > lacZ* Individual, Related to Figure 6 Walking behavior of heterozygous e*lavGS > Aβ42/+* females after two weeks on RU486.

Video S3. Locomotion of *elavGS > Aβ42, azot+/+;+* Individual, Related to Figure 6 Walking trajectories of heterozygous *elavGS > Aβ42, azot+/+;+* females after two weeks on RU486.

**Figure 7:**
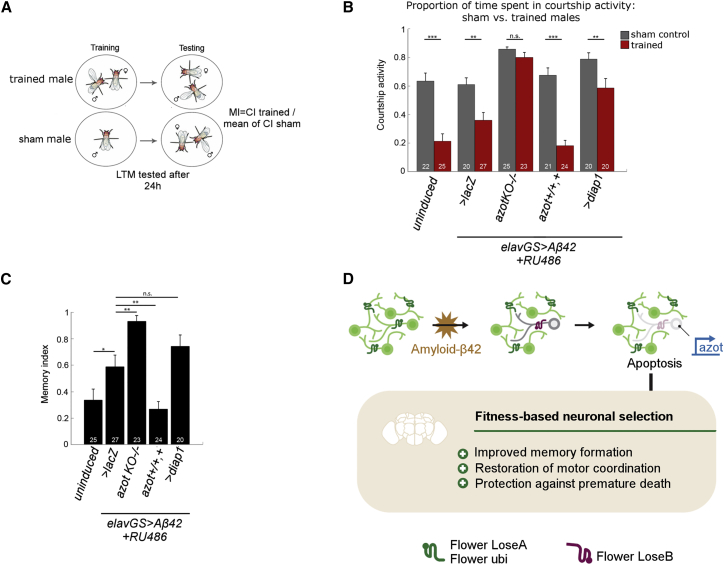
Extra Copy of *azot* Improves Long-Term Memory Formation in Flies Expressing Amyloid-β42 (A) Schematic depicting the courtship suppression assay to measure long-term memory. (B) Graph showing the courtship index of sham males (gray bars) versus trained males (red bars) of the indicated genotypes. Statistical analysis was based on Student’s t test. (C) Graph showing the memory index of the indicated genotypes. Statistical analysis was based on ANOVA. (D) Amyloid-β42-induced neuronal death is mediated by fitness comparison encoded by *flower* and executed by *azot*. Removal of amyloid-β42-compromised neurons via cell competition has a strong net beneficial effect at the organismal level, protecting against motor decline, memory impairment, and premature death. Error bars show SEM, and numbers within the bars indicate the number of individuals tested. ^∗∗∗^p < 0.001, ^∗∗^p < 0.01, ^∗^p < 0.05. See also Figure S6 and Videos S4, S5, S6, S7, and S8.

To assess long-term memory (LTM) formation, we used courtship suppression assays (Keleman et al., 2007, Siegel and Hall, 1979). Courtship conditioning is a form of associative learning by which male flies have to recall that they were previously rejected by a mated-unreceptive female and reduce courtship activity when re-exposed (Figure 7A). Because prolonged *Aβ42* expression resulted in locomotion defects, we reduced *Aβ42* induction by one week to ensure that all naive control males reached a courtship index of 0.6–0.8 (courting 60%–80% of the observation period) (Figure 7B), normally seen in wild-type sham controls (Nichols et al., 2012). Presence of RU486 did not affect LTM formation of *elavGS* flies without the *Aβ42* transgene (data not shown). We measured a significant difference in courtship index between sham and trained males for all genotypes except for *elavGS > Aβ42, azotKO*^−/−^ flies (Figure 7B). One week-induced *elavGS > Aβ42* flies showed impaired LTM formation compared to uninduced flies (Videos S4 and S5), which was strongly aggravated in the absence of *azot* (Figures 7B and 7C; Video S6). Additional expression of > *diap* to block cell death had a detrimental effect on LTM (but not a statistically significant one) (Figure 7C; Video S7). Conversely, introduction of an extra copy of *azot*, which increases the efficiency of cell culling (Merino et al., 2015), was sufficient to restore robust LTM formation in A*β42*-expressing flies (Figures 7C and 7D; Video S8), resulting in significant improvement of memory compared to *elavGS > Aβ42 / > lacZ* flies. These results underline that *azot*-mediated clearance of neurons is beneficial for motor and cognitive functions affected by adult onset of *Aβ42* expression. Moreover, introduction of a single extra copy of *azot* was sufficient to prevent *Aβ42*-induced motor and cognitive decline (Figure 7D).

Video S4. Courtship Activity of Trained e*lavGS > Aβ42 / > lacZ* Flies Induced for One Week on RU486, Related to Figure 7

Video S5. Courtship Activity of Trained e*lavGS > Aβ42 / > lacZ* Flies Raised for One Week on Normal Food (No Induction), Related to Figure 7

Video S6. Courtship Activity of Trained e*lavGS > Aβ42, azotKO*^−/−^ Flies Induced for One Week on RU486, Related to Figure 7

Video S7. Courtship Activity of Trained e*lavGS > Aβ42 / > diap* Fly Induced for One Week on RU486, Related to Figure 7

Video S8. Courtship Activity of Trained elavGS > Aβ42, azot+/+;+ Fly Induced for One Week on RU486, Related to Figure 7

In flies, the mushroom body (MB) is important for learning and memory (Aso et al., 2014). To investigate whether the previously observed memory defects were caused by altered mushroom body architecture, we revealed mushroom body structure using an anti-Fasciclin II (FasII) antibody, which strongly labels the α and β lobes (Crittenden et al., 1998, Fushima and Tsujimura, 2007). We analyzed all genotypes after one week of Aβ42 induction when memory phenotypes were evident, but we did not find strong changes in mushroom body structure. In particular, *elavGS > Aβ42, azotKO*−/− flies did not exhibit severe morphological defects despite the strong memory impairment (Figure S6B). We observed a modest variability among individuals of the same genotype and depicted mild alterations in lobes of the mushroom body, which were comparable among genotypes (Figure S6B). This result suggests that memory differences between genotypes are not a result of mushroom body malformation but rather a consequence of a genetic interaction between Aβ42 and *azot*.

## Discussion

Here, we report that expression of misfolding-prone toxic peptides linked to AD and Huntington’s disease affects neuronal fitness and triggers competition between neurons, leading to increased activation of the Flower^LoseB^ isoform and Azot in *Drosophila* neural tissues. Our results demonstrate that fitness fingerprints are important physiological mediators of neuronal death occurring in the course of neurodegenerative diseases. This mechanism is associated with specific toxic peptides or with particular stages of the neurodegenerative disease, because competition is not elicited by expression of Parkinson-related α-Synuclein, for instance. Our results suggest that the toxic effects of a given peptide correlate directly with the level of neuronal competition and death it induces.

Surprisingly, we found that neuronal death had a beneficial effect against β-amyloid-dependent cognitive and motor decline. This finding challenges the commonly accepted idea that neuronal death is detrimental at all stages of the disease progression. We found that most amyloid-induced neuronal apoptosis is beneficial and likely acts to remove damaged and/or dysfunctional neurons in an attempt to protect neural circuits from aberrant neuronal activation and impaired synaptic transmission.

One curious observation in our study is that Aβ42 induces cell death both autonomously and non-autonomously in clones of the eye disc. Dying cells co-localize with Flower^LoseB^ reporter both inside and outside of GFP-marked clones of the larva. We observed that Aβ42 peptide is secreted to regions outside of clone borders and accumulates at the basal side of the eye disc. The neurons of the eye disc that project their axons into the optic stalk through the basal side of the disc are likely affected by the accumulation of the toxic peptide, explaining the induction of cell death outside of clones.

We observed that blocking apoptosis in Aβ42 expressing flies by either *azot* silencing or overexpression of dIAP1 increases the number of vacuoles in the brains of these flies. This seems to be a counterintuitive observation, because one would expect that a reduction in apoptosis would result in fewer cells being lost and a reduction of neurodegenerative vacuoles. However, this observation can be conciliated with our model: we suspect that less fit neurons have impaired dendritic growth and inhibit the expansion of neighboring neurons. This inhibition would disappear once the unfit neuron is culled, allowing compensatory dendritic growth and neuropil extension.

Introduction of a single extra copy of *azot* was sufficient to prevent *Aβ42*-induced motor and cognitive decline, which may suggest new venues for AD treatment that aim to support elimination of dysfunctional neurons at early stages of AD pathology. For example, in patients at early symptomatic stages, when cognitive impairment is first detected, enhancing physiological apoptotic pathways using Bcl-2 or Bcl-xL inhibitors, or promoting the cell competition pathway described here, may have strikingly beneficial effects.

## STAR★Methods

### Key Resources Table

**Table:** 

REAGENT or RESOURCE	SOURCE	IDENTIFIER
**Antibodies**		

rat anti-ELAV	DSHB	Cat#7E8A10; RRID: AB_528218
polyclonal anti-Myc-tag	Cell Signaling	Cat#2272; RRID: AB_10692100
monoclonal mouse anti-Aβ42	Synaptic Systems	Cat#218 721; RRID: AB_2619923
anti-Fasciclin II	DSHB	Cat#1D4; RRID: AB_528235
chicken anti-GFP	Abcam	Cat#ab13970; RRID: AB_300798
phalloidin Alexa-546	Molecular Probes	Cat#A22283; RRID: AB_2632953
cleaved DCP1	Cell Signaling	Cat#9578; RRID: AB_2721060

**Chemicals and Commercial Assays**		

Terminal Transferase, recombinant (rTdT)	Roche	Cat#3333574001
Biotin-16-dUTP	Roche	Cat#11093070910
RU486 (Mifepristone)	Sigma	Cat#M8046-1G
propidium iodide	Sigma	Cat#P4170
proteostat aggresome dye	Enzo Life Sciences	Cat#ENZ-51035-0025

**Experimental Models: Fly stocks**		

*UAS-Aβ42 (2x)*	Casas-Tinto et al., 2011	N/A
*GMR-Gal4*	Bloomington	BDSC:1104
*elav-GeneSwitch*	Bloomington	BDSC: 43642
*azot{KO; GFP}*	Merino et al., 2015	N/A
*azot{KO; w+}*	Merino et al., 2015	N/A
*azot{KO; hid}*	Merino et al., 2015	N/A
*UAS-diap1*	Bloomington	BDSC: 6657
UAS-f*lower*^*LoseB*^	Rhiner et al., 2010	N/A
*flower*^*LoseA/B*^*RNAi (long hairpin)*	Merino et al., 2013	N/A
*UAS-flower RNAi kk*	VDRC	VDRC: 104993
UAS- *azot RNAi GD*	VDRC	VDRC: 18166
UAS- *azot RNAi kk*	VDRC	VDRC: 102353
*UAS-secEMAP*	Casas-Tintó et al., 2015	N/A
*UAS-Aβ40*	Bloomington	BDSC: 64215
*UAS-httQ0* and *UAS-httQ128*	Lee et al., 2004	N/A
UAS-*αsynWT* and *UAS-αsynA30P*	Bloomington	BDSC: 8146 BDSC: 8147
*flower*^*ubi*^*-YFP,flower*^*LoseA*^*-GFP,flower*^*LoseB*^*-RFP* and flower^ubi^-flag, flower^LoseA^-HA, flower^LoseB^-myc	Yao et al., 2009	N/A
*UAS-lacZ*	Bloomington	BDSC: 3955
*act > y+ > gal4*	Rhiner et al., 2010	N/A

### Contact for Reagent and Resource Sharing

Further information and requests for resources and reagents should be directed to the Lead Contact, Eduardo Moreno (eduardo.moreno@research.fchampalimaud.org).

### Experimental Model and Subject Details

Flies were maintained on standard cornmeal-molasses-agar media and reared at 25°C under 12h alternating light-dark cycles. Stocks used in this study are listed in the Key Resources Table.

### Method Details

#### Generation of flower^LoseB^::mCherry and azot::mCherry reporter

The *flower*^*LoseB*^*::mCherry knock-in* was made by genomic engineering (Baena-Lopez et al., 2013, Huang et al., 2009). The genomic engineering by Huang is a 2-step process consisting of ends-out gene targeting followed by phage integrase *phi31*-mediated DNA integration. A founder knock-out line was established with a genomic deletion of the *flower* locus at position 3L: 1’5816’737-15810028. A *knock-in* construct containing the deleted *flower* locus fused with mCherry after exon 6 (specific for *flower*^*LoseB*^ isoform) was integrated in the KO line. The knock-in construct was done by site directed mutagenesis to remove the stop codon of exon 6 and add a restriction site to clone mCherry. The *knock-out* of *flower* and the *knock-in flower*^*LoseB*^*::mCherry* were proven by PCR. Vectors used for generating the *flower*^*LoseB*^*::mCherry* were as following: *pGX-attP*: (Knock-out vector), *pGEM-T* (used for the site directed mutagenesis and insertion of mCherry) and *pGEattB*^*GMR*^ (*Knock- in* vector).

Primers used were the following: AAGCGGCCGCAGCAGCAACAACAGCAGCAACG and AAGCGGCCGCACCGTTCAATATGCAGGCGGC (5′ arm *flower* amplification), GGAGATCTGGATGATTCCTGAGCTGCGGTAT and AACTGCAGATGGGGACACCTAAAGAGGCACC (3′ arm *flower* amplification),

AACTATATTGGGCCGGCCAAGCTAACCGAATGCAAGAGGAACCGGAACCTA and GCATTCGGTTAGCTTGGCCGGCCCAATATAGTTTCTCACTAAAAATATATGCTTGC (mutagenesis primers), TAGGGCCGGCCATGGTGTCCAAGGGCGAAG and ATGGCCGGCCCTTATTTATACAGCTCGTCCATGC (mCherry amplification), ACATAGATCTATAAAAGCTTTCAATGTACACAAATTTG and AGCTGGCGCGCCAAAAAGCATGCCCCACAATAGTTAC (for *flower* KI).

The *azot::mCherry* reporter was generated by fusion PCR to combine *mCherry* coding sequence to *azot* genomic region, including 2430bps upstream of the start codon, the full *azot* exon and 175 bp at the 3′ UTR. *azot* genomic region was amplified from the Bac clone CH321-21G13 (http://pacmanfly.org/) using the following primers: TTGCTTAGACTGTGGCCAGAG and CTCTTCGCCCTTGGACACCATTCGCATTGTCATCATGTTGACGA for 5′ region and *azot* exon; GACATCTTCTCGCCCAGGTTG and ATGGACGAGCTGTATAAATAACCTCCATGTGAGTACTCGTA for 3′ UTR; GAGATCTCGACGTTCATACGGACGGACAGGCAGACGGAAGGAC and ACTGCATATAACATGCGCGAGA for the promoter region of *azot*. *mCherry* was amplified from *c5_stable2_neo* vector with primers TCAACATGATGACAATGCGAATGGTGTCCAAGGGCGAAGAG and ACGAGTACTCACATGGAGGTTATTTATACAGCTCGTCCATG. The final construct was obtained by two rounds of fusion PCRs (first with primers TAGGCGCGCCCCGCTCATTGTTTCCAAAGTGATTTTC and GCCGCTAGCGTATGAACGTCGAGATCTCGG; second with primers ACTGCATATAACATGCGCGAGA and TAGGCGCGCCCCGCTCATTGTTTCCAAAGTGATTTTC) and was cloned in *pGEattBGMR* with the restriction sites *NheI* and *AscI*.

#### Immunohistochemistry and image acquisition

Wandering third instar larvae were collected and eye imaginal discs dissected. For clone induction, larvae were given a heat shock at 37°C 48h or 72h before dissection. For pupal dissections, white prepupae (0hr) were collected and maintained at 25°C for 40h. Dissections were performed in chilled PBS, samples were fixed for 30min in formaldehyde (4% v/v in PBS) and permeabilized with PBT 0,4%Triton.

The primary antibodies and fluorescent reagents used in this study are listed in the Key Resources Table. TUNEL staining (Roche) was performed according to the supplier’s protocol and modified as previously (Lolo et al., 2012). For detection of protein inclusions, brains were fixed and permeabilized as described above and incubated for 1h30min with the proteostat aggresome dye (Enzo Life Sciences) before mounting. For the necrosis assay, brains were dissected in PBS 1X and incubated for 30min at room temperature with 10 μg/ml propidium iodide (PI) (Sigma-Aldrich) in Schneider medium, following by washing and standard fixation ((Liu et al., 2014, Yang et al., 2013)). Samples were mounted in Vectashield (Vectorlab) and imaged on a Leica confocal SP5 or a Zeiss LSM 880 using a 20X dry objective or a 40X oil objective.

#### Longevity Assays and brain morphology

To minimize disturbing neural development and reduce differences on the genetic background, the RU486-inducible *GeneSwitch* system was employed (Osterwalder et al., 2001, Roman et al., 2001). The stock solution of RU486 (Mifepristone, Sigma, prepared in 80% ethanol) was diluted in MiliQ water to a final concentration of 100 μM and 300 μL of the diluted solution was added to the surface of the fly food and allowed to dry at room temperature for 48-36h (Poirier et al., 2008). For the mock solution, 80% ethanol was diluted 10X in water. For survivorship analysis, newly eclosed flies were transferred to bottles and allowed to mate for 2 days at 25°C (He and Jasper, 2014, Linford et al., 2013). Females were then sorted into groups of 15-20 (more than 100 flies in total were used per genotype) and placed at 29°C into vials containing standard food supplemented either with RU468 or mock solution. Flies were transferred to new food every 2-3 days and dead/censored animals were counted. For brain morphology analysis, males were subjected to the same protocol, aged at 29°C until the required stage and dissected. To quantify number of *azot*-expressing cells with *azot{KO;GFP},* newly eclosed males were collected and kept at 25°C to age for 5, 15 or 30 days.

#### Behavioral assays

Detailed protocol and further description of the Buridan’s arena can be found elsewhere (Colomb and Brembs, 2014, Colomb et al., 2012). Shortly, 2 weeks-old females, kept in a 12/12 hours light/dark regime, were raised on standard or RU486-containing food. The day before measurements, flies were CO_2_ -anaesthetized (max 5 min) and their wings were cut with surgical scissors at two thirds of their length. For recordings, flies were placed in the center of the Buridan’s platform in a dark room. The walking activity of each individual fly was recorded for 5 minutes with the Buritrack software (http://buridan.sourceforge.net). Individual tests were re-initalized when flies jumped from the platform or exhibited grooming behavior. Walking behavior was analyzed with the CeTrAn software V4 (https://github.com/jcolomb/CeTrAn/releases/tag/v.4).

Long-term memory (Courtship suppression assay): The repeat training courtship assay was used to assess 24 hour long-term memory formation as published previously (Fitzsimons et al., 2013). Briefly, a training session was conducted by coupling individual males with a freshly mated female for a period of seven hours, while sham males were housed alone and served as controls to verify that courtship activity of a specific genotype was intact. Males were induced on RU486 food for one week, which was previously shown to be sufficient to induce the *elavGS* driver and elicit *Aβ42* expression in fly heads (Rogers et al., 2012). After 24 hours, all males, trained and sham, were coupled with new mated females and courtship activity was measured over a period of ten minutes as the percentage of time spent courting (courtship index, CI)(Reza et al., 2013). A memory index (MI) was then calculated as the ratio between the CI of every trained male and the mean value of the CI of the sham males of the same genotype. A range of scores between zero and one was obtained, with zero indicating good memory and one indicating memory similar to a sham control(Ejima and Griffith, 2007), e.g., no memory. Normal memory is generally characterized by a MI of 0.5-0.7 (Fitzsimons et al., 2013). Collected flies were flipped onto fresh food every two days and kept at 25°C in a 12 hr light/dark cycle. *elavGS > Aβ* flies on standard food served as uninduced control. In all experiments, the experimenter was blind to the genotype of the flies. Experiments were performed under ambient light at 25°C with 65%–70% relative humidity and recorded for 10min using a camcorder (Sony Handycam HDR PJ410).

### Quantification and Statistical Analysis

Image quantification was done with Fiji. The number of positive cells in the adult brain for DCP1, TUNEL, Flower^LoseB^::mCherry, Flower^LoseB^::RFP or Azot::mCherry was counted on 40-μm-wide maximum projections including the anterior part of the optic lobe. Noise signal was removed using a Gaussian blur filter (sigma = 1) and/or applying a background subtraction (rolling = 20). GFP expressing cells in *azot{KO;GFP*} flies were assumed to be GFP-positive particles wider than 9pixels on a 25 μm-thick projection (showing a 141 μm^2^ field) of the optic lobe. Measure of death induction in eye imaginal discs was done by counting the number of TUNEL positive particles in 10 μm-thick maximum projections. Spaces between phalloidin staining with an area > 25 μm2 were assumed to be neurodegenerative vacuoles. Presence of vacuoles was quantified two weeks after eclosion at a 10 μm deep ventral plane located in the central brain (next to the mushroom body).

#### Survival curves

For statistical analysis, a log-rank test (Mantel Cox) was applied to determine significant differences between survival curves.

Walking behavior data was analyzed by an ANOVA model, which was validated posthoc with Tukey-Anscombe plot and QQ plot of the residuals. p values were calculated comparing all experimental genotypes with each other and corrected for multiple testing using Holm’s method (Holm, 1979). The variables distance, activity time, and turning angle were chosen for analysis based on previous test experiments.

Courtship suppression assay, raw data was subjected to arcsine transformation in order to obtain a normal distribution and the memory indexes of each genotype were subjected to a one-way ANOVA followed by Bonferroni and Holm’s correction by comparing genotypes to *elavGS > Aβ* induced controls. When comparing only two genotypes, the Student’s t test (two-tailed, unpaired) was used. Significance was set at p < 0.05.

The distribution of the number of positive cells (for DCP1, Flower^LoseB^::mCherry, Flower^LoseB^::RFP or Azot::mCherry) in the optic lobes of adult flies was analyzed for statistical significant differences between groups with a Kruskal-Wallis test and a Dunn’s test was applied for multiple comparisons between genotypes. The number of brain vacuoles per hemisphere was analyzed for homogeneity between genotypes with a Levene test and p values were calculated with one way ANOVA and a Dunnett’s posthoc test. To determine statistical differences between genotypes for the number of TUNEL positive cells in eye discs, a one-way ANOVA test, followed by a Dunnett’s posthoc were applied. When only two groups were compared and data did not follow a normal distribution assessed by d’Agostino-Pearson omnibus test, statistical significance was accessed with a Mann-Whitney U non-parametric test (for example in the quantification of TUNEL positive cells in the adult brain, GFP positive cells in the optic lobe and clone area). The number of PH3 positive cells was analyzed with an unpaired t test with Welch’s correction. All graphs are displayed as mean ± standard error.

### Data and Software Availability

Buritrack (http://buridan.sourceforge.net), CeTrAn V4 (https://github.com/jcolomb/CeTrAn/releases/tag/v.4), Fiji (https://fiji.sc/), and GraphPad prim 6 (https://www.graphpad.com/scientific-software/prism/).

## Supplemental Information

Document S1. Figures S1–S6

Document S2. Article plus Supplemental Information
