# Supplementary material for: Culling Less Fit Neurons Protects against Amyloid-β-Induced Brain Damage and Cognitive and Motor Decline
Source: Cell Rep. 2018 Dec 26;25(13):3661–3673.e3. doi: 10.1016/j.celrep.2018.11.098 (PMC6315112; doi:10.1016/j.celrep.2018.11.098)
Supplement: Document S1. Figures S1–S6 [file mmc1.pdf]

Cell Reports, Volume *25*

Supplemental Information

Culling Less Fit Neurons Protects against Amyloid-β-Induced Brain Damage and Cognitive and Motor Decline

Dina S. Coelho, Silvia Schwartz, Marisa M. Merino, Barbara Hauert, Barbara Topfel, Colin Tieche, Christa Rhiner, and Eduardo Moreno

Supplemental Information

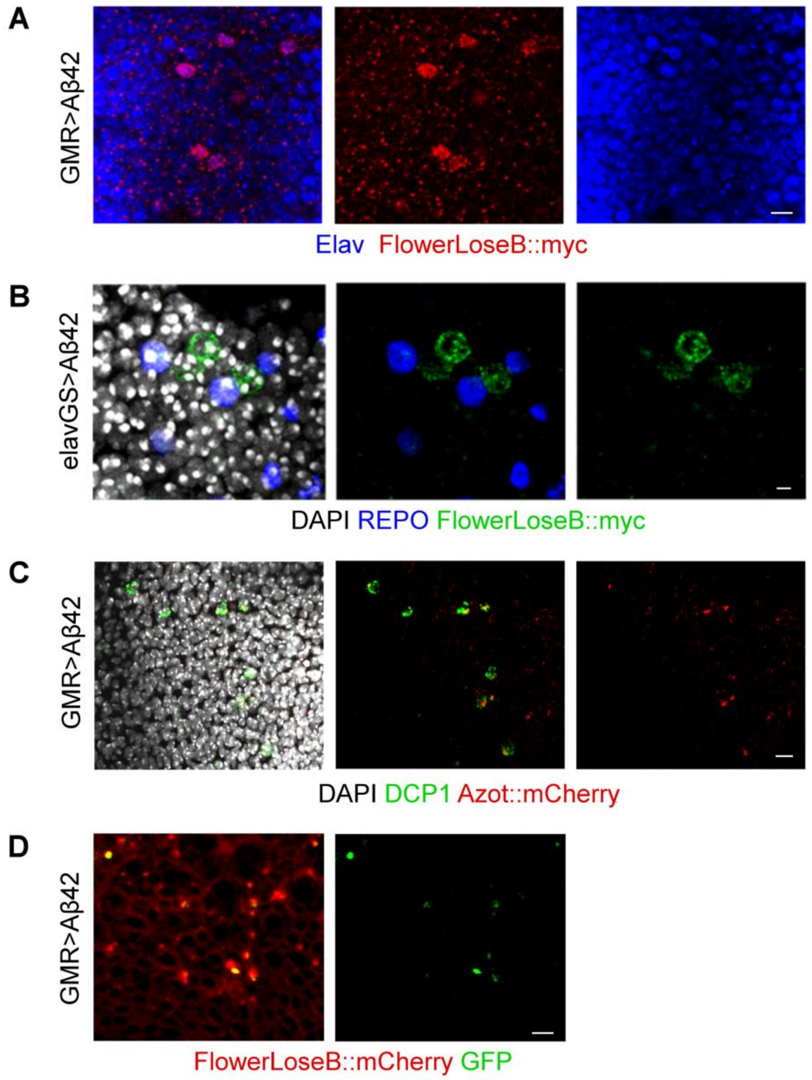

Figure S1. Neurons but not glial cells co-localize with Flower^{LoseB}. Related to Figure 1 and Figure 4.

(A) The Myc-tag (red) of the *flower^{ubi}-flag, flower^{LoseA}-HA, flower^{LoseB}-myc* reporter co-localizes with neuronal cells marked by Elav (blue) in the optic lobe of *GMR>Aβ42* individuals. Scale bar: 5µm**.**

(B) The Myc tag (green) of the flower^{ubi}-flag, flower^{LoseA}-HA, flower^{LoseB}-myc reporter does not co-localize with REPO positive cells corresponding to glia (blue) in the optic lobe of *GMR>Aβ42* individuals. DAPI is in white. Scale bar: 2µm.

(C) A subset of DCP1 labeled apoptotic cells *(*green) are also Azot::mCherry positive (red) in eye imaginal discs of *GMR>Aβ42* third instar larva. DAPI is in white. Scale bar: 5µm.

(D) All GFP signal derived of the *azot{KO;GFP}* construct (green) co-localizes with Flower^{LoseB}::mCherry cells (red), but not all Flower^{LoseB}::mCherry expression co-localizes with GFP of azot{KO;GFP} in eye imaginal discs of *GMR>Aβ42* third instar larva. Scale bar: 5µm.

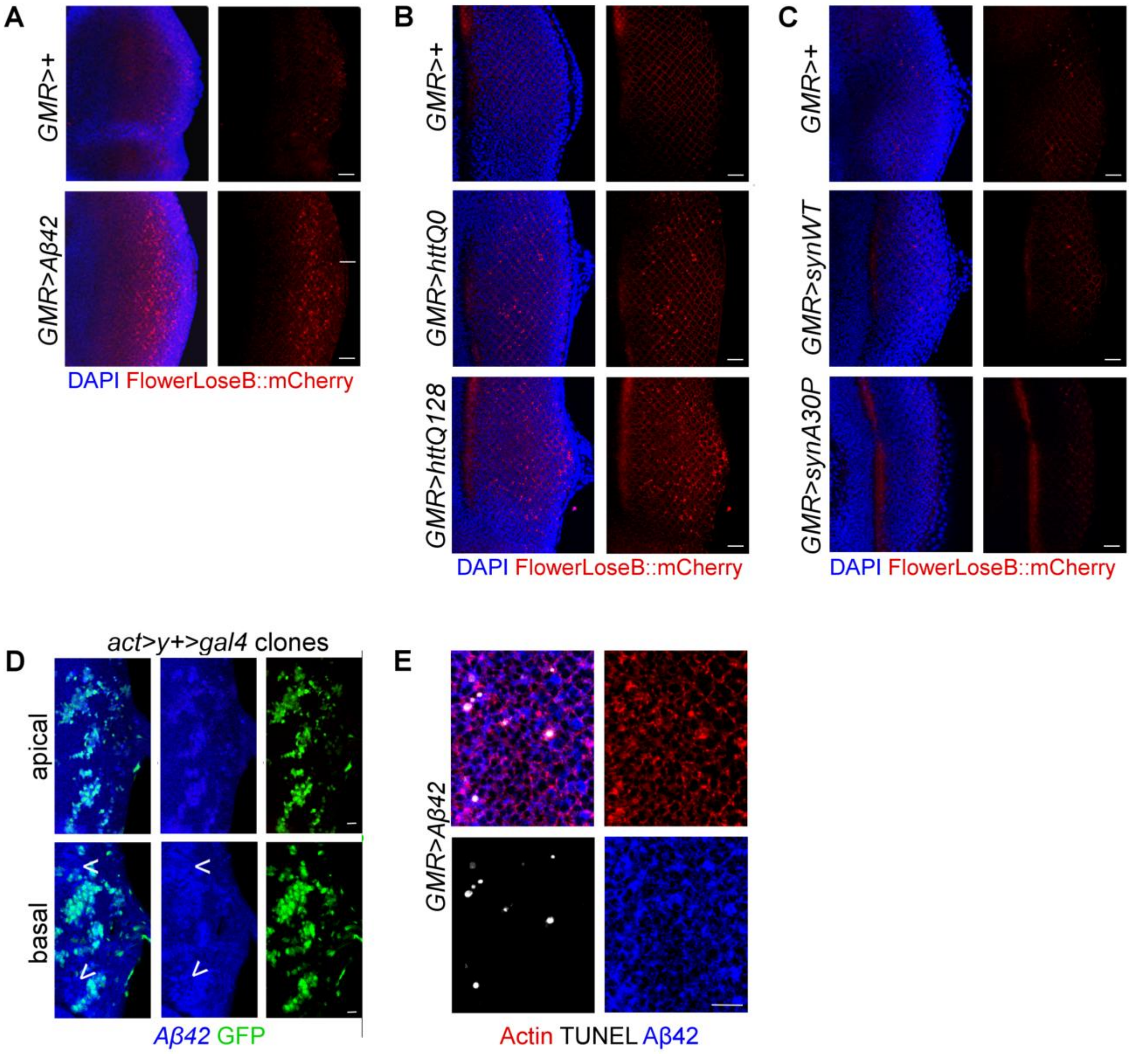

Figure S2 Flower^{loseB}::mCherry reporter (red) is strongly induced by overexpression of *httQ128* in eye imaginal discs. Related to Figure 2 and Figure 3

(A) The Flower^{loseB}::mCherry reporter (red) is strongly upregulated in *GMR*>Aβ42 eye imaginal discs of third instar larva comparing to *GMR*>+ only control eye discs; the nuclear marker DAPI is shown in blue. Scale bar: 20 µm

(B) The Flower^{loseB}::mCherry reporter (red) is strongly expressed in *GMR>httQ128* eye imaginal discs*,* moderately expressed in *GMR>httQ0* eye discs but is not detected in *GMR*>+ only controls; the nuclear marker DAPI is shown in blue. Scale bar: 20 µm

(C) The Flower^{loseB}::mCherry reporter (red) is not expressed in eye imaginal discs of *GMR>α-synWT* and *GMR> α-synA30P* larva. DAPI is shown in blue. Scale bar: 20 µm

(D) Aβ42 localization (blue) in the apical side and basal side of eye discs 24h after the induction of *act*>*y*+>*gal4* clones (green). Arrows show Aβ42 accumulation out of the clone borders, evident on the basal side. Scale bar:10µm.

(E) TUNEL labeling of cell death (white) detected in the eye disc of *GMR>Aβ42* larva. Actin is shown in red and Aβ42 peptide recognized by a specific antibody in blue. Scale bar: 10µm

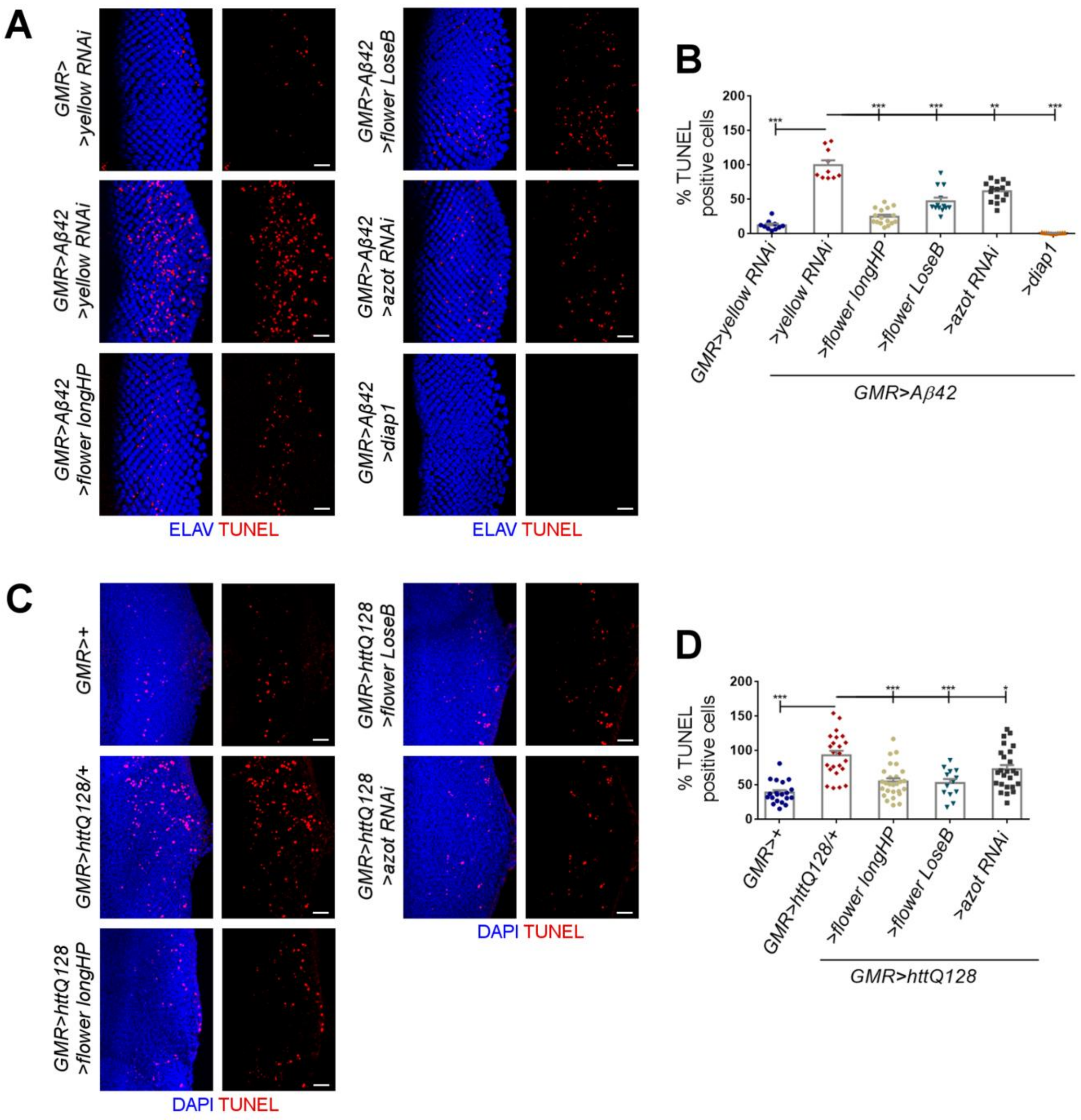

Figure S3 *flower* and *azot* mediate cell death induced by toxic proteins related to neurodegenerative diseases Related to Figure 4

(A) Eye imaginal discs of third instar larva showing TUNEL labeling (red) of apoptotic cells, representative for each genotype. The nuclear marker ELAV is in blue. Scale bar: 20µm cells

(B) Quantification of the number of TUNEL-positive cells per eye disc, assuming the number of apoptotic cells in *GMR>Aβ42*/ *yellow RNAi* as 100%.

(C) Eye imaginal discs of third instar larva showing TUNEL labeling (red) of apoptotic cells, representative for each genotype. The nuclear marker DAPI is in blue. Scale bar: 20 µm

(D) Quantification of the number of TUNEL-positive cells per eye disc, assuming the number of apoptotic cells in *GMR>httQ128* / + as 100%.

Error bars show SEM. ***P value<0,001. **P value<0,01. *P value<0,05.

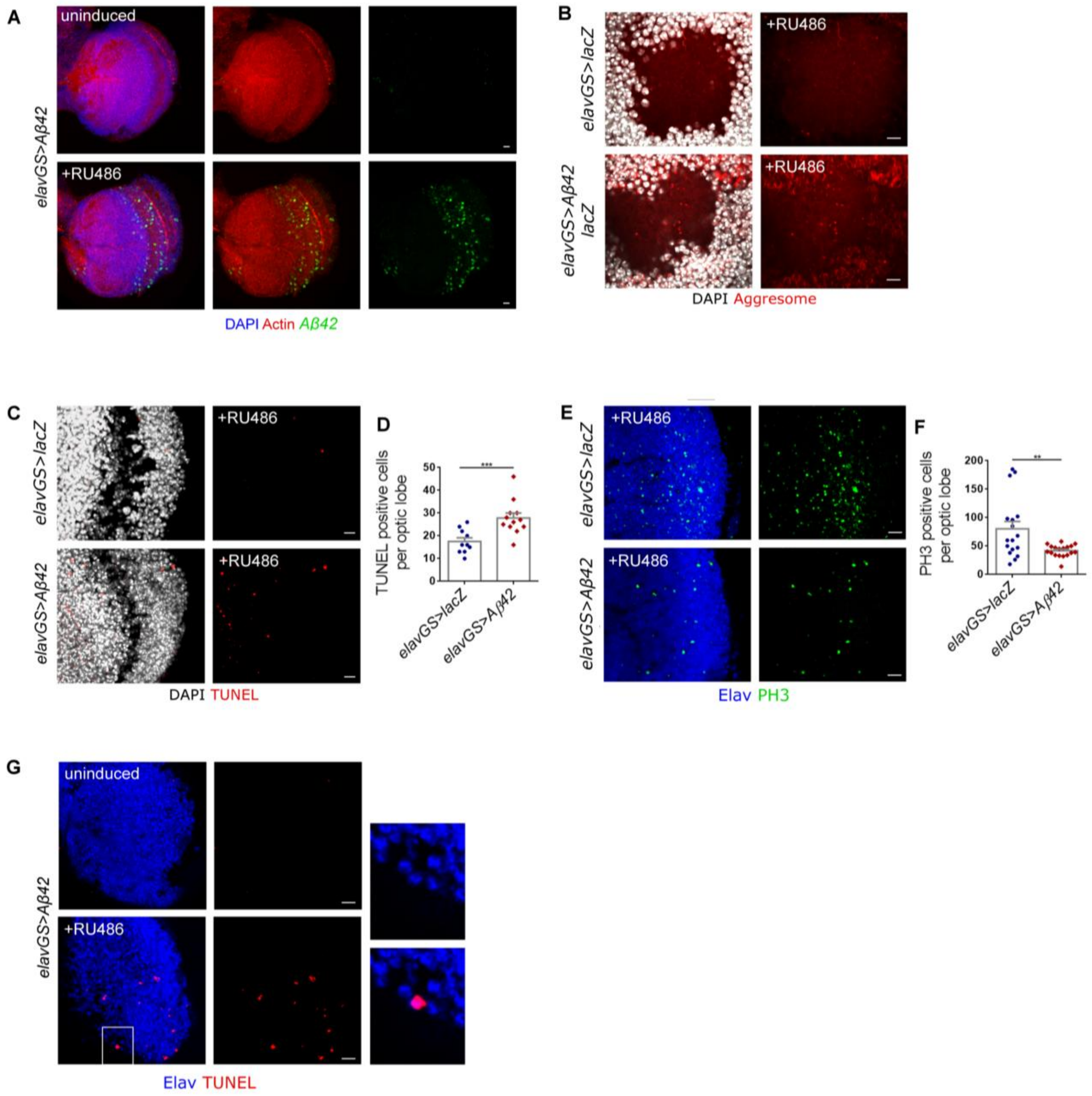

Figure S4. Conditional expression of human Aβ42 in the adult brain of *Drosophila* causes neuronal death and the formation of inclusion bodies Related to Figure 5.

(A) Conditional expression of human Aβ42 peptide in the *Drosophila* adult brain under the control of the *elav-GeneSwitch (elavGS)* driver in the presence of RU486. Aβ42 aggregates (green) are detected in the optic lobe of induced *elavGS>Aβ42* flies but not in uninduced controls. Actin is in red and nuclei are in blue. Scale bar:10 µm.

(B) Inclusion bodies formed by aggregated proteins (or aggresomes - in red) detected by the proteostat aggresome dye located in the mushroom body calyx of *elavGS>lacZ* or e*lavGS>Aβ42 / >lacZ* flies raised on food supplemented with RU486. DAPI in white. Scale bar: 5µm

(C,D) Quantification and representative images of apoptotic cells in the optic lobe of *elavGS>lacZ* or *elavGS>Aβ42* flies fed on RU486-supplemented food. Apoptotic cells are labelled by TUNEL (red) and DAPI is in white. Scale bar: 5µm.

(E) PH3 staining (green) in the optic lobe of e*lavGS>lacZ* or elavGS>Aβ42 flies treated with RU486**.** Elav is in blue. Scale bar: 10µm.

(F) Quantification of the number of PH3 positive cells per optic lobe of the indicated genotypes at two weeks old. 10µm

(G) TUNEL-positive cells (red) in the optic lobes of *elavGS>Aβ42* males, uninduced or fed with RU486 for 10days. Neurons are marked by ELAV in blue. Scale bar: 10µm.

Error bars show standard error mean. ***P value<0,001, **P value<0,01. *P value<0,05.

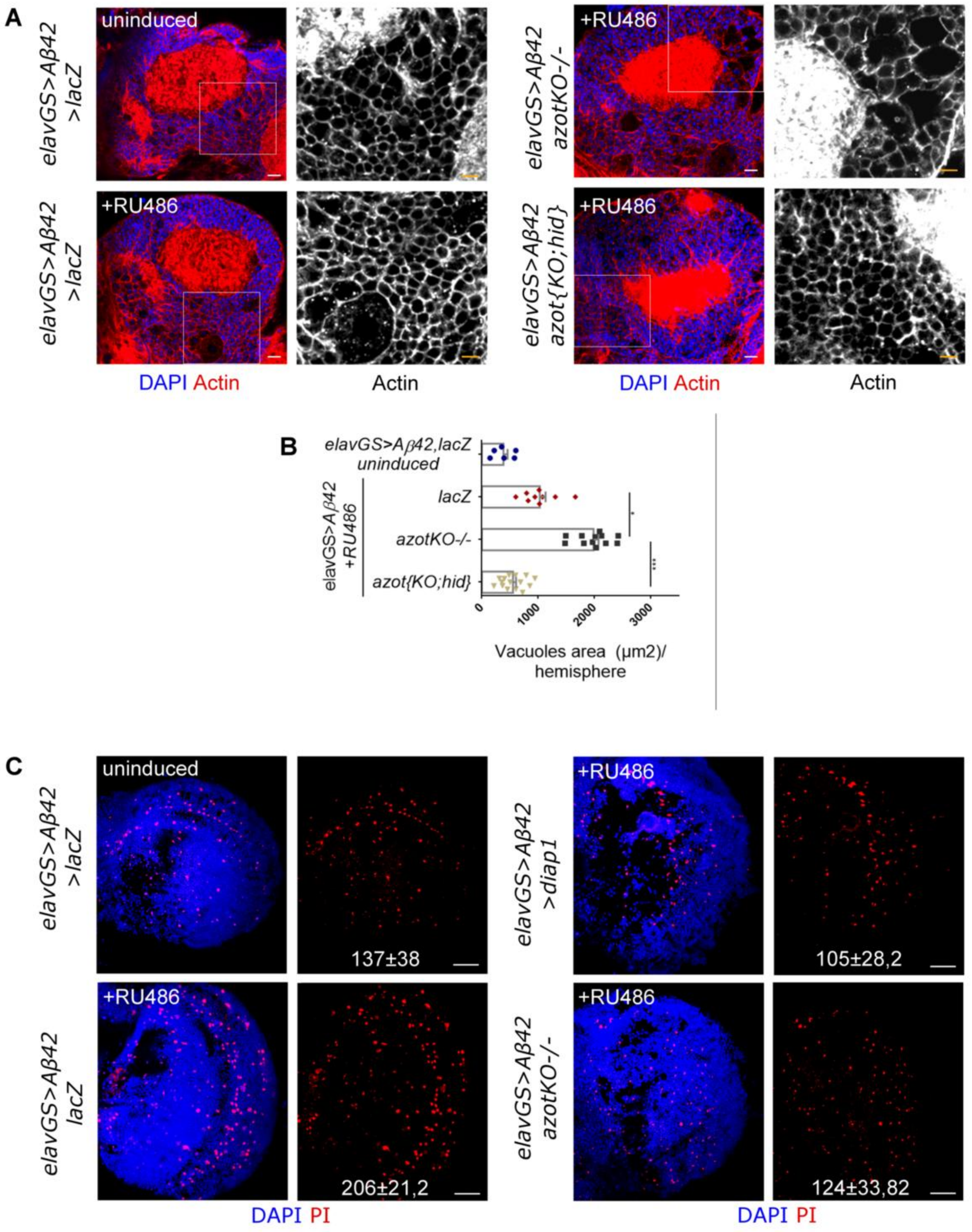

Figure S5. Azot is exclusively dedicated to cell death during neurodegeneration Related to Figure 5.

(A) Ventral plane of the central brain, focusing on the region adjacent to the mushroom body to show degenerative vacuoles present in the following genotypes: uninduced *elavGS>Aβ42/ >lacZ*, induced *elavGS>Aβ42/>lacZ* (+RU486), induced *elavGS>Aβ42, azotKO-/-* (+RU486), induced *elavGS>Aβ42/azot{KO;hid}* (+RU486). Phalloidin binding actin filaments is in red and DAPI shows nuclei in blue. Scale bar: 20µm in color pictures or 10µm in grayscale insets.

(B) Mean of the total area occupied by vacuoles (in $µm^2$) per brain section (at a 10µm deep plane). Error bars show SEM. ***P value<0,001, *P value<0,05.

(C) Image displaying necrotic cells marked by PI (red) in the optic lobe of two weeks old males, representative for each genotype: uninduced *elavGS>Aβ42/ >lacZ*, induced *elavGS>Aβ42/ >lacZ* (+RU486), induced *elavGS>Aβ42, azotKO*$^{-/-}$ (+RU486) and induced *elavGS>Aβ42/ >diap1*. The average number (and standard deviation of the mean) of positive PI cells per optic lobe for each genotype is shown at the bottom of the image. DAPI is in blue. Scale bar: 20µm.

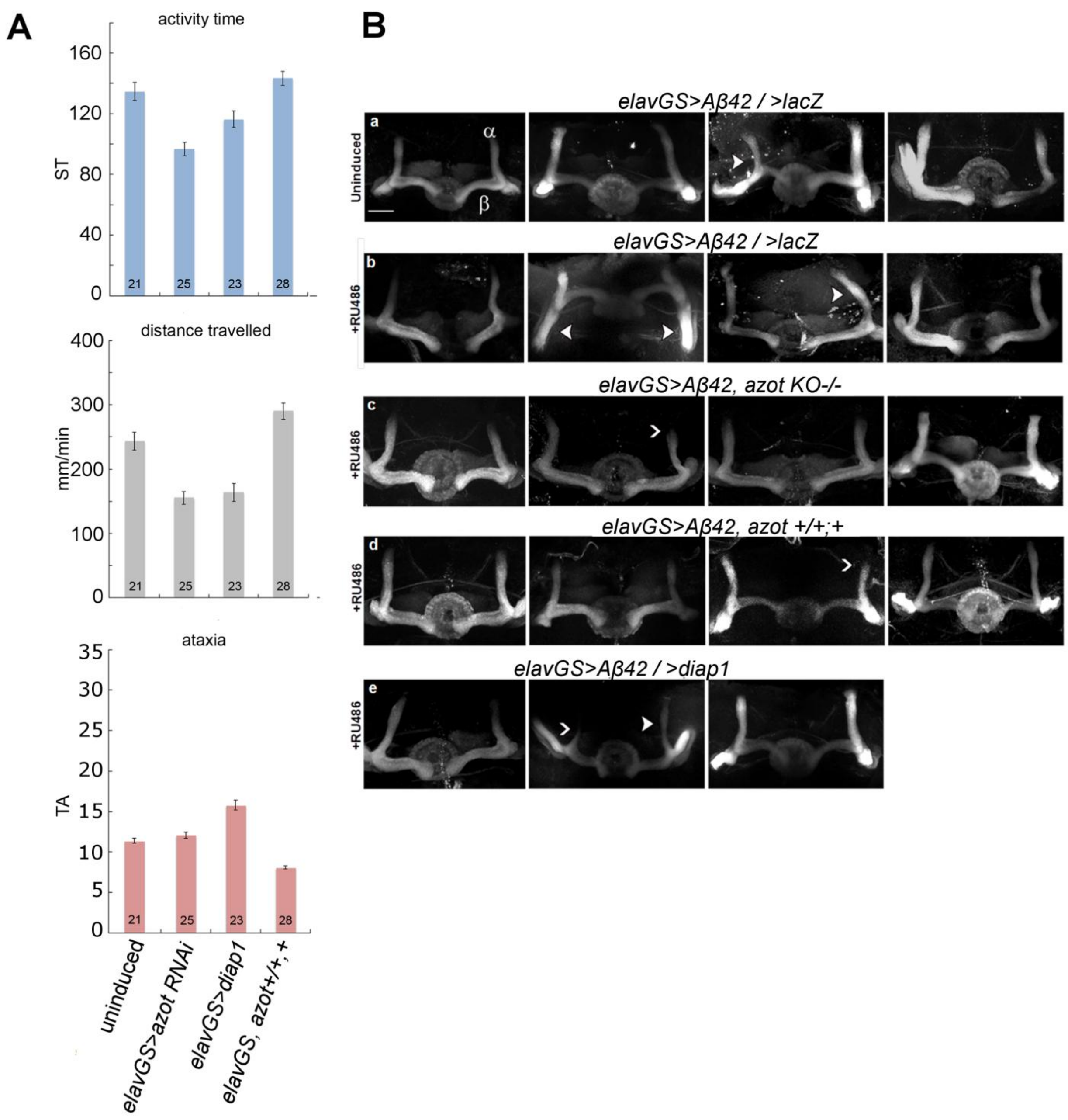

Figure S6. Morphology of the mushroom body (α and β lobes). Related to Figure 6 and 7.

(A) Graphs depicting activity time in seconds (using a speed threshold, ST)(Colomb et al., 2012), distance walked in mm/min and median turning angle (TA) in degrees. Parameters were calculated from individual walks of two weeks-old heterozygous flies raised on RU486 for each genotype. Error bars show S.E.M., numbers indicate number of individual flies tested.

(B) Immunohistochemistry with anti-FasII on whole-mount brains depicting the morphology of the mushroom body lobes in one week-old males of the following genotypes: uninduced *elavGS>Aβ42/ >lacZ*, induced *elavGS>Aβ42/>lacZ*, induced *elavGS>Aβ42, azotKO-/-*, induced *elavGS>Aβ42, azot+;+/+* and induced *elavGS>Aβ42/ >diap1*). A mild phenotypic variability of the mushroom body lobes was observed consisting of the following traits: outgrowth and guidance defects indicated by a white arrow and irregular shape indicated by a white arrowhead. Images are projections of confocal z-stacks. Scale bar = 50 μm.
